# Antenatal betamethasone enhanced the detrimental effects of postnatal dexamethasone on hyperoxic lung and brain injuries in newborn rats

**DOI:** 10.1371/journal.pone.0221847

**Published:** 2019-08-30

**Authors:** Young Eun Kim, Won Soon Park, Dong Kyung Sung, So Yoon Ahn, Yun Sil Chang

**Affiliations:** 1 Department of Pediatrics, Samsung Medical Center, Sungkyunkwan University School of Medicine, Seoul, Korea; 2 Stem Cell and Regenerative Medicine Institute, Samsung Medical Center, Seoul, Korea; Centre Hospitalier Universitaire Vaudois, FRANCE

## Abstract

**Purpose:**

To determine the effects of antenatal betamethasone and/or postnatal dexamethasone administration on hyperoxic lung and brain injuries in newborn rats.

**Methods:**

Newborn Sprague-Dawley rats were divided into five experimental groups: normoxia-vehicle-vehicle group, hyperoxia-vehicle-vehicle group, hyperoxia-betamethasone-vehicle group, hyperoxia-vehicle-dexamethasone group, and hyperoxia-betamethasone-dexamethasone group according to (i) whether rats were exposed to normoxia or hyperoxia after birth to postnatal day (P) 14, (ii) whether antenatal betamethasone (0.2mg/kg) or vehicle was administered to pregnant rats at gestation days 19 and 20, and (iii) whether three tapering doses of dexamethasone (0.5, 0.3, 0.1mg/kg per day) or vehicle were administered on P5, 6 and 7, respectively. The lungs and brains were harvested for histological and biochemical analyses at P8 and P14.

**Results:**

Postnatal dexamethasone but not antenatal betamethasone significantly enhanced hyperoxia-induced reduction in body weight gain and alveolarization and increased lung terminal deoxynucleotidyl transferase dUTP nick end labeling (TUNEL) positive cells both at P8 and P14, transiently increased hyperoxia-induced reductions in brain weight gain and angiogenesis, and increase in brain TUNEL-positive cells at P8 but not at P14. Co-administration of antenatal betamethasone significantly enhanced dexamethasone-induced impairments in alveolarization both at P8 and P14, transient increases in lung and brain oxidative stresses, and increases in brain TUNEL-positive cells at P8 but not at P14.

**Conclusion:**

Although postnatal dexamethasone but not antenatal betamethasone alone significantly increased hyperoxic lung and brain injuries, co-administration of antenatal betamethasone significantly enhanced the detrimental effects of postnatal dexamethasone on hyperoxic lung and brain injuries in newborn rats.

## Introduction

Bronchopulmonary dysplasia (BPD) is a chronic lung disease that occurs in premature infants receiving prolonged ventilator support and high oxygen supplementation [1]. BPD survivors typically suffer from long-term injuries not only to the lungs such as asthma or chronic obstructive pulmonary disease [2] but also to the brain with resulting neuro-functional deficits such as developmental delay and cerebral palsy in later life [3–5]. In concordance with the clinical findings, hyperoxia-induced injury to the lung and brain are closely correlated [6, 7]. Inhaled nitric oxide [8] or intratracheal transplantation of mesenchymal stem cells [6] simultaneously attenuated both hyperoxic lung and brain injuries in newborn rats, revealing a close relationship with the extent of such attenuation between the lung and brain. Collectively, the close association between the extent of BPD and concurrent brain injury suggest that any clinically effective pulmo-protective therapies are also neuroprotective in premature infants.

Antenatal and/or postnatal steroids remain clinically available and effective interventions for lung maturation. However, various preclinical and clinical studies [9, 10] have suggested that the impacts of antenatal and/or postnatal steroids on BPD are controversial with studies showing no or some benefit. Moreover, there is a concern in the use of perinatal steroids for causing neurodevelopmental harm [11]. Therefore, further preclinical and clinical studies are necessary to balance the potential benefits and harms of antenatal and/or postnatal steroid therapy.

In the present study, we used a newborn rat model of chronic exposure to high oxygen to induce neonatal hyperoxic lung and brain injuries [6], mimicking clinical BPD and associated brain injury in preterm infants. We tried to determine whether antenatal and/or postnatal steroids could simultaneously attenuate or increase both lung and brain injuries, and whether the extents of such attenuation or increase in the lung and brain tissues were correlated in hyperoxic newborn rats.

## Materials and methods

### Animal model

All animal procedures were approved by the Institutional Animal Care and Use Committee of Samsung Biomedical Research Institute (Seoul, Korea), and the animals were housed in an Assessment and Accreditation of Laboratory Animal Care International accredited facility. Dam rats were maintained with an alternating 12-h light/dark cycle under constant room humidity and temperature. We monitored the condition of rat pups on a weekly basis and twice per day on a daily basis, particularly for the 14 days after hyperoxia exposure. In this study, we used humane endpoint as the earliest indicator in an animal experiment of pain or distress that could be used to avoid or limit pain and distress by taking actions such as humane euthanasia. For humane endpoint, operationally defined scoring system was approved by IACUC. Total scores of ≥ 5 or score 3 in any single category were arbitrarily defined as a humane endpoint. Humane endpoints consisted of body weight growth (1: slower growth than normal rats, 2: growth arrest, 3: weight loss), responsiveness (1: delayed but appropriate response, 2: delayed and null response, 3: no response), and appearance (1: rough hair coat, 2: porphyrin staining, 3: sustained abnormal posture or dilated pupil). Throughout the experimental period, no rat pups reached a humane endpoint.

**Fig 1:**
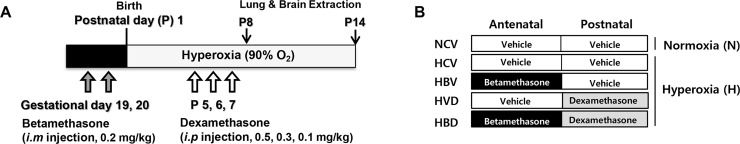
Experimental protocol. (A) Schematic outline of the animal model of antenatal and postnatal steroids-exposed newborn rat pups under hyperoxia. (B) Experimental study groups. NCV, normoxia control with antenatal vehicle and postnatal vehicle; HCV, hyperoxia control with antenatal vehicle and postnatal vehicle; HBV, hyperoxia with antenatal betamethasone and postnatal vehicle; HVD, hyperoxia with antenatal vehicle and postnatal dexamethasone; and HBD, hyperoxia with antenatal betamethasone and postnatal dexamethasone.

As experimental animals, timed-pregnant Sprague-Dawley rats (Oriental Bio Co, Seongnam, Korea) were divided into two groups: one group was antenatally administered betamethasone and the other was administered vehicle. All mother rats spontaneously delivered pups at gestational day (E) 22 within 3h of time-interval and none of the pups were stillborn. Within 10h of birth, antenatal betamethasone-treated and normal rat pups were divided into hyperoxia (90% O_2_) or normoxia (21% O_2_) groups for 14 days, as in our previous studies [12–15]. Hyperoxia-exposed rats were subdivided based on exposure or non-exposure to postnatal dexamethasone. Based on the experimental conditions—i) normoxia (N) or hyperoxia (H), ii) antenatal betamethasone (B) or its vehicle (C), and iii) postnatal dexamethasone (D) or its vehicle (V)—the five groups were termed as NCV, HCV, HBV, HCD and HBD groups, as shown in Fig 1.

For administration of antenatal steroids, two doses of betamethasone (0.2mg/kg per day) (betamethasone sodium phosphate; Huons, Seongnam, Korea) were intramuscularly administered to timed-pregnant rats at E19 and E20. After birth, for administration of postnatal steroids, a tapering course of dexamethasone (0.5, 0.3, 0.1mg/kg per day) (dexamethasone disodium phosphate; Jeil Pharmaceutical, Seoul, Korea) was intraperitoneally administered from P5 to P7. Control rat pups were administered an equal volume of vehicle (normal saline) in the same manner. To investigate the effects of the steroid on the hyperoxic lung and brain at P8 and P14, lung and brain tissues were obtained after transcardiac perfusion with ice-cold normal saline under deep pentobarbital (Entobar, Hanlim Pharmaceutical C7-8o., Seoul, Korea) anesthesia (60 mg/kg, intraperitoneal).

In the present study, total 12 litters were used, 2 litters for NCV, 5 litters for antenatal betamethasone and subsequent HBV and HBD groups, and 5 litters for antenatal vehicle and subsequent HCV and HVD groups, respectively. There were no stillbirths, and the litter sizes at birth ranged from 11 to 14 (n = 13 ± 1 in NCV, n = 13± 2 in antenatal steroid and subsequent HBV+HBD, n = 12 ± 2 in antenatal vehicle and subsequent HCV+HVD, respectively, p>0.05). Two litters, one with antenatal betamethasone and one with antenatal vehicle exposure, were exposed to 14 days of hyperoxia simultaneously, and each litter was randomly allocated for HBV or HBD, and HCV or HVD, respectively, and half of them in each group were randomly sacrificed at p8, and the rest were randomly sacrificed at p14 for histological and biochemical analyses, respectively. The number of harvested lung and brain samples was evenly distributed for histological analyses at P8 (n = 7, 5, 7, 6 and 7 in the NCV, HCV, HBV, HVD and HBD groups, respectively) and at P14 (n = 6, 5, 6, 6 and 5 in the NCV, HCV, HBV, HVD and HBD groups, respectively). For biochemical analyses, the number of tissue samples were also evenly distributed at P8 (n = 7, 6, 7, 6 and 7 in the NCV, HCV, HBV, HVD and HBD groups, respectively) and at P14 (n = 6, 5, 5, 6 and 6 in the NCV, HCV, HBV, HVD and HBD groups, respectively).

### Tissue preparation

The whole brain, extracted from the olfactory bulbs to the cerebellum, was weighed on an electronic scale accurate to two decimal points (Scout Pro, OHAUS, Parsippany, NJ, USA). For histological observation, the tissues were fixed with 10% buffered formalin at room temperature, embedded in paraffin blocks, and sliced into 4-micrometer-thick sections. For biochemical observation, the tissues were snap-frozen, stored at -80°C, and homogenized shortly before analyses.

### Morphometric measurement of lung alveolarization

For lung morphometric measurement, the lungs were exposed by thoracotomy and transcardiac perfusion with ice cold phosphate buffered saline. The lungs were fixed by the tracheal instillation of 4% paraformaldehyde solution at a constant inflation pressure of 25 cm H_2_O. The trachea was then ligated, and the lungs were removed, and immersed in the same fixative overnight at room temperature. The fixed lungs were embedded in paraffin, sectioned at 4μm, and then stained with hematoxylin and eosin. The mean linear intercept (MLI; mean interalveolar distance) [16], mean alveolar volume (MAV) [17], and radial alveolar count (RAC) [18] were measured as described in our previous study [12].

### Enzyme-linked immunosorbent assay

After the tissues were homogenized and centrifuged, protein contents in each supernatant were adjusted to the same concentration. The levels of interleukin (IL)-1α, IL-6, tumor necrosis factor (TNF)-α, and vascular endothelial growth factor (VEGF) were measured using Milliplex MAP ELISA Kit following the manufacturer’s protocol (Millipore, Billerica, MA, USA).

### TUNEL assay

To observe cell death in the lung and brain sections, terminal deoxynucleotidyl transferase dUTP nick end labeling (TUNEL) was conducted using DeadEnd Fluorometric TUNEL System kit (G3250, Promega, Madison, WI, USA). After counter-staining and mounting with Vector shield mounting medium containing DAPI (Vector Laboratories, Burlingame, CA, USA), the number of TUNEL-positive cells was counted in 6 non-overlapping fields of the lung and brain (periventricular zone, +0.48 to +0.2 mm/Bregma; hippocampal dentate gyrus, -3.60 to -3.08 mm/Bregma; cortex, -3.60 to -3.80 mm/Bregma).

### Immunohistochemistry

We histologically investigated lung angiogenesis and brain myelination by immunostaining. To quantify endothelial density in the lungs, lung sections were stained with von Willebrand factor (vWF) antibody (1:250; A0082, DAKO, Carpentaria, CA, USA) as a primary antibody and stained with Alexa Fluor 568 goat-anti rabbit (1:500; A11036, Invitrogen, Carlsbad, CA, USA) as a secondary antibody. In the brains (cingulate white matter and lateral corpus callosum, -3.60 to -3.08 mm/Bregma), myelinated nerve fibers were stained with myelin basic protein (MBP) antibody [SMI-94] (1:500; ab24567, Abcam, Cambridge, UK) as a primary antibody and Alexa Fluor 488 goat-anti mouse (1:500; A11001, Invitrogen) as a secondary antibody.

### Oxidative stress measurement

In lung and brain lysates, oxidized protein levels were detected using Protein Carbonyl Western Blot Detection kit (Cosmo Bio, Tokyo, Japan) according to the manufacturer’s protocol. The lysate samples were separated on Novex Bolt 4–12% Bis-Tris gels (Life Technologies, Carlsbad, CA, USA) and then transferred onto polyvinylidene fluoride membranes (iBlot Gel Transfer Stacks PVDF Regular, Invitrogen). The carbonyl groups in the protein side chains were derivatized to 2,4-dinitrophenylhydrazine, and then detected by western blot analysis using anti-2,4-dinitrophenol antibody.

### Statistical analysis

All data are presented as the mean ± standard error of the mean (SEM). Survival rates were compared by Kaplan–Meier analysis. Comparisons among groups were made using mixed model with “group” as fixed and “mother rat” as random effect for the consideration of pregnancies from which subjects originated, followed by Tukey-Kramer post hoc test. Spearman correlation coefficient was calculated for lung and brain injury correlation analyses. All data were analyzed with SPSS Statistics 18 software (PASW 18, SPSS Inc., Chicago, IL, USA). *P*-values less than 0.05 were considered statistically significant.

## Results

### Survival rate, body and brain weight

**Fig 2:**
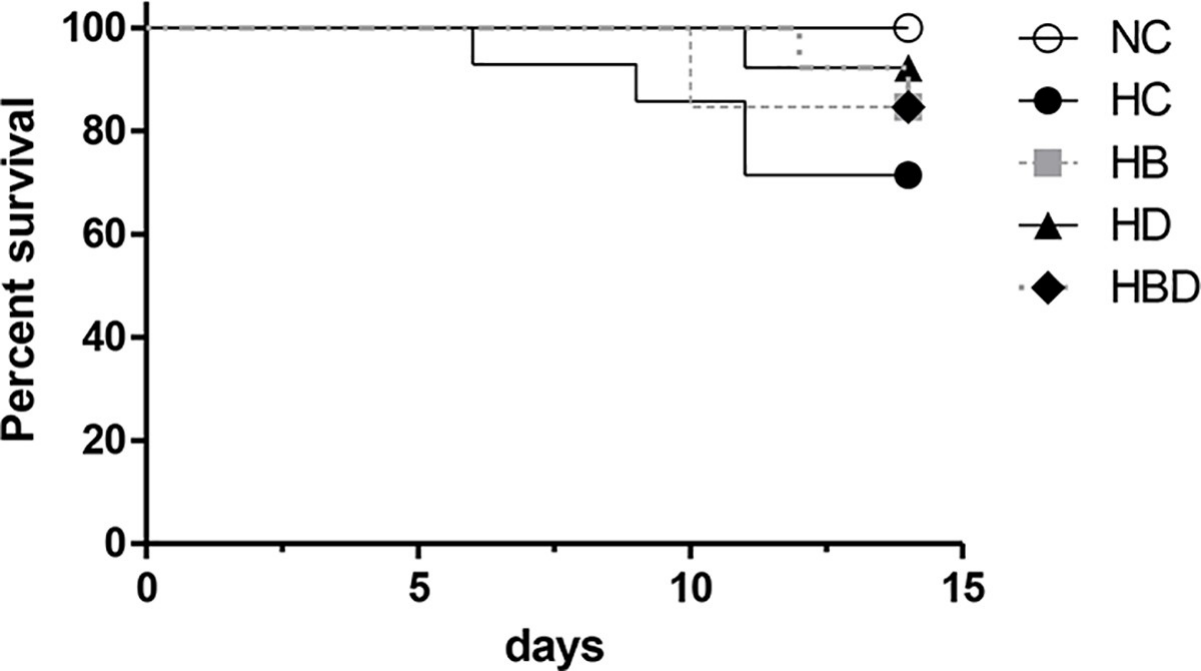
Survival rate after exposure to hyperoxia and steroids. Time course of survival in each experimental group for 14 days after birth (n of survival/total = 12/12, 10/14, 11/13, 12/13 and 11/13 in NCV, HCV, HBV, HVD and HBD, respectively). NCV, normoxia control with antenatal vehicle and postnatal vehicle; HCV, hyperoxia control with antenatal vehicle and postnatal vehicle; HBV, hyperoxia with antenatal betamethasone and postnatal vehicle; HVD, hyperoxia with antenatal vehicle and postnatal dexamethasone; and HBD, hyperoxia with antenatal betamethasone and postnatal dexamethasone.

Exposure to hyperoxia reduced the survival rate at P14 from 100% in the NCV group to 71%, 85%, 92% and 85% in the HCV, HBV, HVD, and HBD groups, respectively, however the reduced survival rates were not significantly different from the rate of the NCV group (Fig 2).

Although birth weight did not differ significantly between the study groups, both body and brain weights at P8 were significantly lower in the HVD and HBD groups than in the NCV, HCV, and HBV groups (Table 1). At P14, body weight was significantly lower in the HCV and HBV groups and the lowest in the HVD and HBD groups than in the NCV group. Brain weight was significantly lower in the HCV, HBV, HVD, and HBD groups compared to that in the NCV group.

**Table 1:** Changes in body weight and brain weight after exposure to hyperoxia and steroids.

	Postnatal day 1	Postnatal day 8		Postnatal day 14	
	Birth weight	Body weight	Brain weight	Body weight	Brain weight
**NCV**	6.65 ± 0.171	16.3 ± 0.25	0.62 ± 0.010	27.7 ± 0.24	1.11 ± 0.020
**HCV**	6.85 ± 0.122	16.9 ± 0.27	0.60 ± 0.010	25.8 ± 0.43 *	1.01 ± 0.022 *
**HBV**	6.46 ± 0.132	16.2 ± 0.36	0.59 ± 0.012	26.0 ± 0.64 *	1.01 ± 0.023 *
**HVD**	6.81 ± 0.127	12.0 ± 0.33 *, #, $	0.54 ± 0.008 *, #, $	23.1 ± 0.45 *, #, $	0.99 ± 0.015 *
**HBD**	6.53 ± 0.154	12.4 ± 0.27 *, #, $	0.53 ± 0.009 *, #, $	23.1 ± 0.51 *, #, $	0.98 ± 0.016 *

Birth weight was measured at postnatal day (P) 1 (n = 26, 21, 27, 23, and 25 in the NCV, HCV, HBV, HVD, and HBD groups, respectively). Body weight and brain weight measured at P8 (n = 14, 11, 16, 11, and 14 in the NCV, HCV, HBV, HVD, and HBD groups, P14 (n = 12, 10, 11, 12 and 11 in NCV, HCV, HBV, HVD, and HBD groups, respectively). Data are given as mean ± SEM.

* P < 0.05 vs. NCV

# P < 0.05 vs. HCV

$ P < 0.05 vs. HBV. NCV, normoxia control with antenatal vehicle and postnatal vehicle; HCV, hyperoxia control with antenatal vehicle and postnatal vehicle; HBV, hyperoxia with antenatal betamethasone and postnatal vehicle; HVD, hyperoxia with antenatal vehicle and postnatal dexamethasone; and HBD, hyperoxia with antenatal betamethasone and postnatal dexamethasone.

### Lung morphological changes

**Fig 3:**
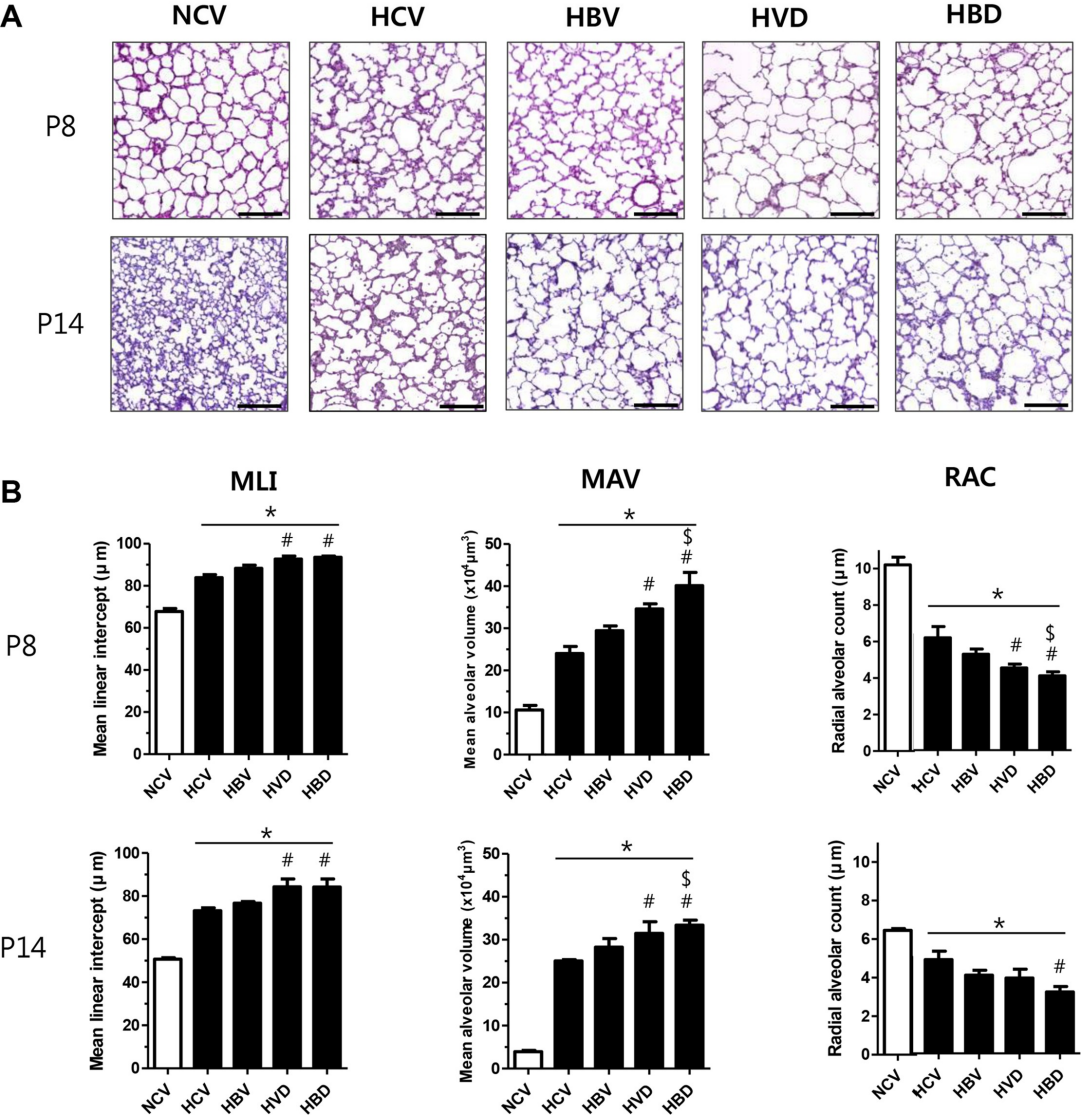
Morphological changes in the lungs following exposure to hyperoxia and steroids. (A) Hematoxylin and eosin (H&E)-stained lung sections showing representative morphology of P8 and P14 rat lungs. (B) Mean linear intercept (μm), mean alveolar volume (×10^4^μm^3^), and radial alveolar count as markers of the degree of alveolarization were measured at P8 (n = 7, 5, 6, 6, and 7 in NCV, HCV, HBV, HVD, and HBD groups, respectively) and at P14 (n = 6, 5, 6, 6, and 5 in NCV, HCV, HBV, HVD, and HBD groups, respectively). Data are given as mean ± SEM. * *P* < 0.05 vs. NCV; # *P* < 0.05 vs. HCV; $ *P* < 0.05 vs. HBV. NCV, normoxia control with antenatal vehicle and postnatal vehicle; HCV, hyperoxia control with antenatal vehicle and postnatal vehicle; HBV, hyperoxia with antenatal betamethasone and postnatal vehicle; HVD, hyperoxia with antenatal vehicle and postnatal dexamethasone; and HBD, hyperoxia with antenatal betamethasone and postnatal dexamethasone.

Representative light microscopic photomicrographs showing histopathological differences between the study groups at P8 and P14 are shown in Fig 3A. While small and uniform alveoli were observed in the NCV group, the HCV group displayed fewer, larger and heterogeneously sized alveoli. In the morphometric analyses, significantly increased MLI and MAV and reduced RAC, indicating impaired alveolarization, were observed in the HCV group compared to those in the NCV group. These values were significantly increased in the HVD group but not in the HBV group, and significantly more enhanced in the HBD group both at P8 and P14 (Fig 3B).

### Lung cell death and angiogenesis

**Fig 4:**
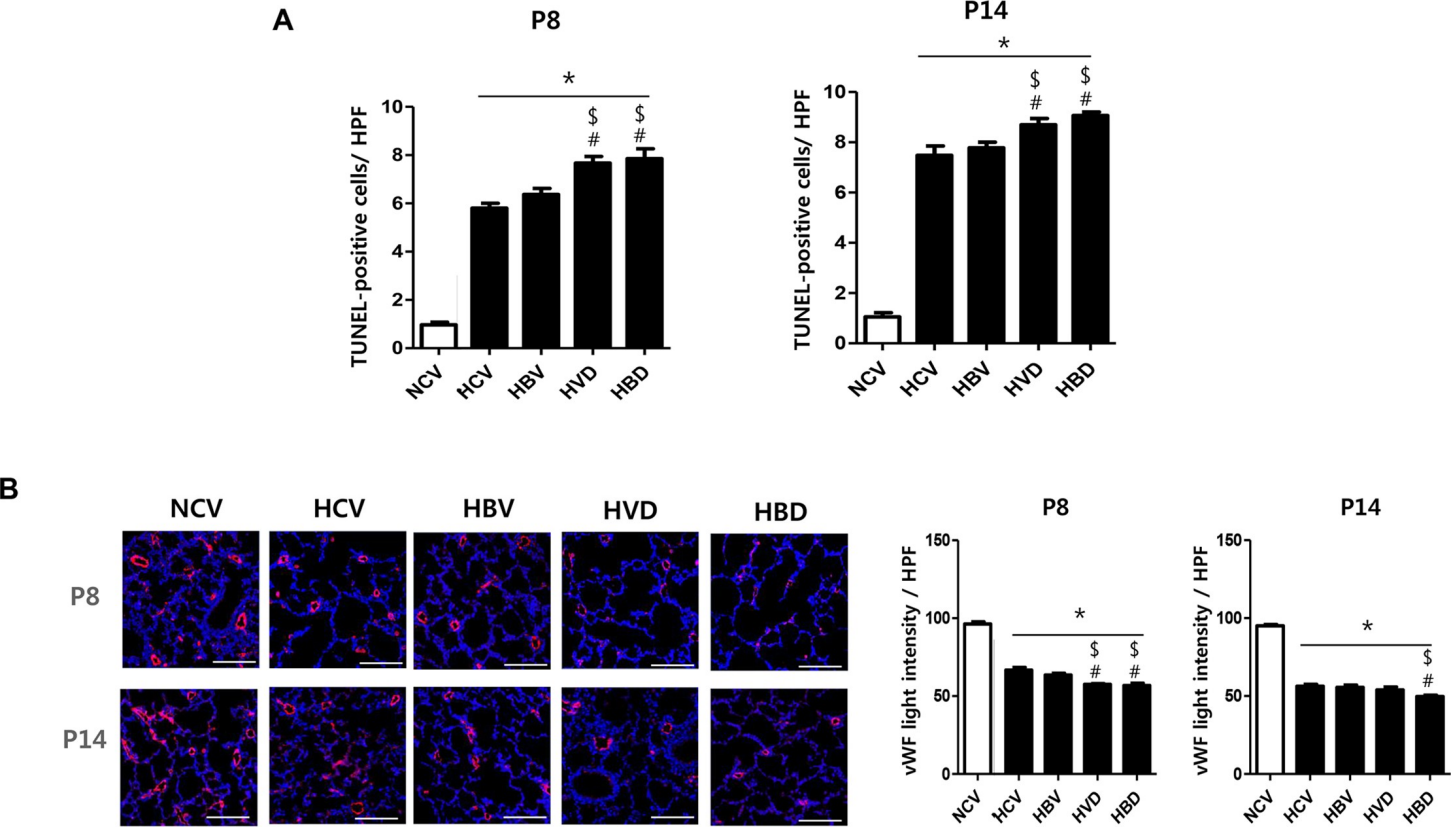
Histological evaluations of lung cell death and angiogenesis in hyperoxia and steroid-exposed lungs. (A) Representative photographs of terminal deoxynucleotidyl transferase dUTP nick end labeling (TUNEL; green)-stained lung sections and average numbers of TUNEL-positive cells per high-power field (HPF) at P8 and P14. (B) The representative photographs of von Willebrand factor (vWF; red) stained lung sections and average intensities of vWF-positive endothelial cells per HPF at P8 and P14. (A–B) Nuclei were counter-stained with 4ʹ,6-diamidino-2-phenylindole (DAPI; blue) (scale bar, 100 μm) (n = 7, 5, 6, 6 and 7 in NCV, HCV, HBV, HVD and HBD at P8, respectively) (n = 6, 5, 6, 6 and 5 in NCV, HCV, HBV, HVD and HBD at P14, respectively). Data are given as mean ± SEM. * *P* < 0.05 vs. NCV; # *P* < 0.05 vs. HCV; $ *P* < 0.05 vs. HBV. NCV, normoxia control with antenatal vehicle and postnatal vehicle; HCV, hyperoxia control with antenatal vehicle and postnatal vehicle; HBV, hyperoxia with antenatal betamethasone and postnatal vehicle; HVD, hyperoxia with antenatal vehicle and postnatal dexamethasone; and HBD, hyperoxia with antenatal betamethasone and postnatal dexamethasone.

The number of TUNEL-positive cells in lungs from HCV group was significantly higher than that in lungs from the NCV group; this hyperoxia-induced increase in TUNEL- positive cells was significantly increased both in HVD and HBD groups but not in the HBV group both at P8 and P14 (Fig 4A).

The intensities of vWF-positive endothelial cells indicative of angiogenesis were significantly reduced in the HCV group compared to that in the NCV group both at P8 and P14. This hyperoxia-induced reduction in lung angiogenesis was significantly increased in both the HVD and HBD groups but not in the HBV group at P8 (Fig 4B). At P14, the hyperoxia-induced reduction in lung angiogenesis was significantly more reduced in HBD group than the HCV and HBV groups.

### Lung cytokines and vascular endothelial growth factor

The levels of IL-1α, IL-6, and TNF-α in the lung were significantly higher in the HCV group than in the NCV group, and the hyperoxia-induced increase in IL-6 and TNF-α but not in IL-1α were significantly increased in the HBV, HVD, and HBD groups both at P8 and P14 (Table 2). The lung VEGF levels in the HCV, HBV, HVD and HBD groups were significantly lowered compared to those in the NCV group both at P8 and P14 (Table 2).

**Table 2:** Inflammatory cytokine and vascular endothelial growth factor (VEGF) levels in lungs exposed to hyperoxia and steroids.

	**Postnatal day 8**			
	**IL-1a**	**IL-6**	**TNF-a**	**VEGF**
**NCV**	9.0 ± 0.58	7.9 ± 0.70	9.3 ± 0.47	85.1 ± 1.67
**HCV**	25.5 ± 1.11 *	20.0 ± 1.18 *	21.3 ± 1.31 *	49.3 ± 2.00 *
**HBV**	29.8 ± 1.20 *	25.0 ± 0.86 *, #	27.0 ± 1.03 *, #	48.8 ± 2.06 *
**HVD**	29.2 ± 1.39 *	26.4 ± 1.12 *, #	27.0 ± 0.86 *, #	50.6 ± 2.06 *
**HBD**	29.5 ± 1.29 *	27.2 ± 1.22 *, #	28.5 ± 0.95 *, #	49.8 ± 2.61 *
				
	**Postnatal day 14**			
	**IL-1a**	**IL-6**	**TNF-a**	**VEGF**
**NCV**	8.5 ± 0.43	8.3 ± 0.67	8.3 ± 0.76	94.0 ± 2.18
**HCV**	27.8 ± 0.96 *	23.6 ± 0.65 *	24.3 ± 1.02 *	45.2 ± 1.52 *
**HBV**	29.8 ± 1.72 *	30.0 ± 1.48 *, #	30.4 ± 1.03 *, #	42.4 ± 3.47 *
**HVD**	31.0 ± 1.20 *	30.6 ± 1.31 *, #	31.8 ± 0.80 *, #	42.63 ± 1.63 *
**HBD**	27.3 ± 0.99 *	30.5 ± 1.02 *, #	31.8 ± 1.70 *, #	42.83 ± 1.99 *

Levels of inflammatory cytokines, such as IL-1α, IL-6, and TNF-α, and VEGF were measured at P8 (n = 7, 6, 7, 6, and 7 in the NCV, HCV, HBV, HVD, and HBD groups, respectively) and at P14 (n = 6, 5, 5, 6 and 6 in NCV, HCV, HBV, HVD and HBD, respectively) rat lungs. Data are given as mean ± SEM.

* P < 0.05 vs. NCV

# P < 0.05 vs. HCV. NCV, normoxia control with antenatal vehicle and postnatal vehicle; HCV, hyperoxia control with antenatal vehicle and postnatal vehicle; HBV, hyperoxia with antenatal betamethasone and postnatal vehicle; HVD, hyperoxia with antenatal vehicle and postnatal dexamethasone; and HBD, hyperoxia with antenatal betamethasone and postnatal dexamethasone.

### Brain cell death and myelination

**Fig 5:**
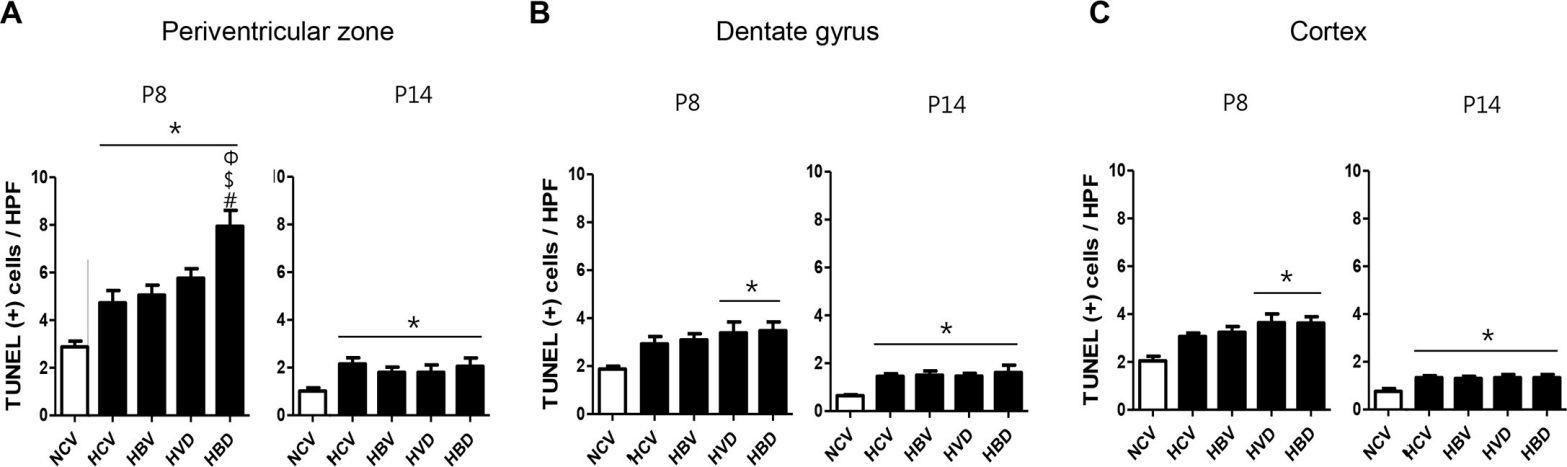
Brain cell death in the periventricular zone, hippocampal dentate gyrus and cortex exposed to hyperoxia and steroids. Representative micrographs of TUNEL-stained periventricular zone, hippocampal dentate gyrus, and cortex in P8 rat brains are shown. The brain tissues were counter-stained with DAPI (4ʹ,6-diamidino-2-phenylindole; blue). The number of TUNEL-positive cells per high-power field (HPF) was counted in P8 and P14 rat brains (scale bar, 100 μm) (n = 7, 5, 6, 6 and 7 in NCV, HCV, HBV, HVD and HBD at P8, respectively) (n = 6, 5, 6, 6 and 5 in NCV, HCV, HBV, HVD and HBD at P14, respectively). Data are given as mean ± SEM. * *P* < 0.05 vs. NCV; # *P* < 0.05 vs. HCV; $ *P* < 0.05 vs. HBV; Φ *P* < 0.05 vs. HVD. NCV, normoxia control with antenatal vehicle and postnatal vehicle; HCV, hyperoxia control with antenatal vehicle and postnatal vehicle; HBV, hyperoxia with antenatal betamethasone and postnatal vehicle; HVD, hyperoxia with antenatal vehicle and postnatal dexamethasone; and HBD, hyperoxia with antenatal betamethasone and postnatal dexamethasone.

The number of TUNEL-positive cells was significantly higher in the HCV group than in the NCV group both at P8 and P14 in the periventricular zone and at P14 but not at P8 in the dentate gyrus and cortex (Fig 5A–5C). The hyperoxia-induced increase in TUNEL-positive cells was significantly but transiently increased in the HBD but not in the HBV and HVD groups in the periventricular zone, and in both HVD and HBD groups but not in the HBV group in the dentate gyrus and cortex only at P8 but not at P14.

**Fig 6:**
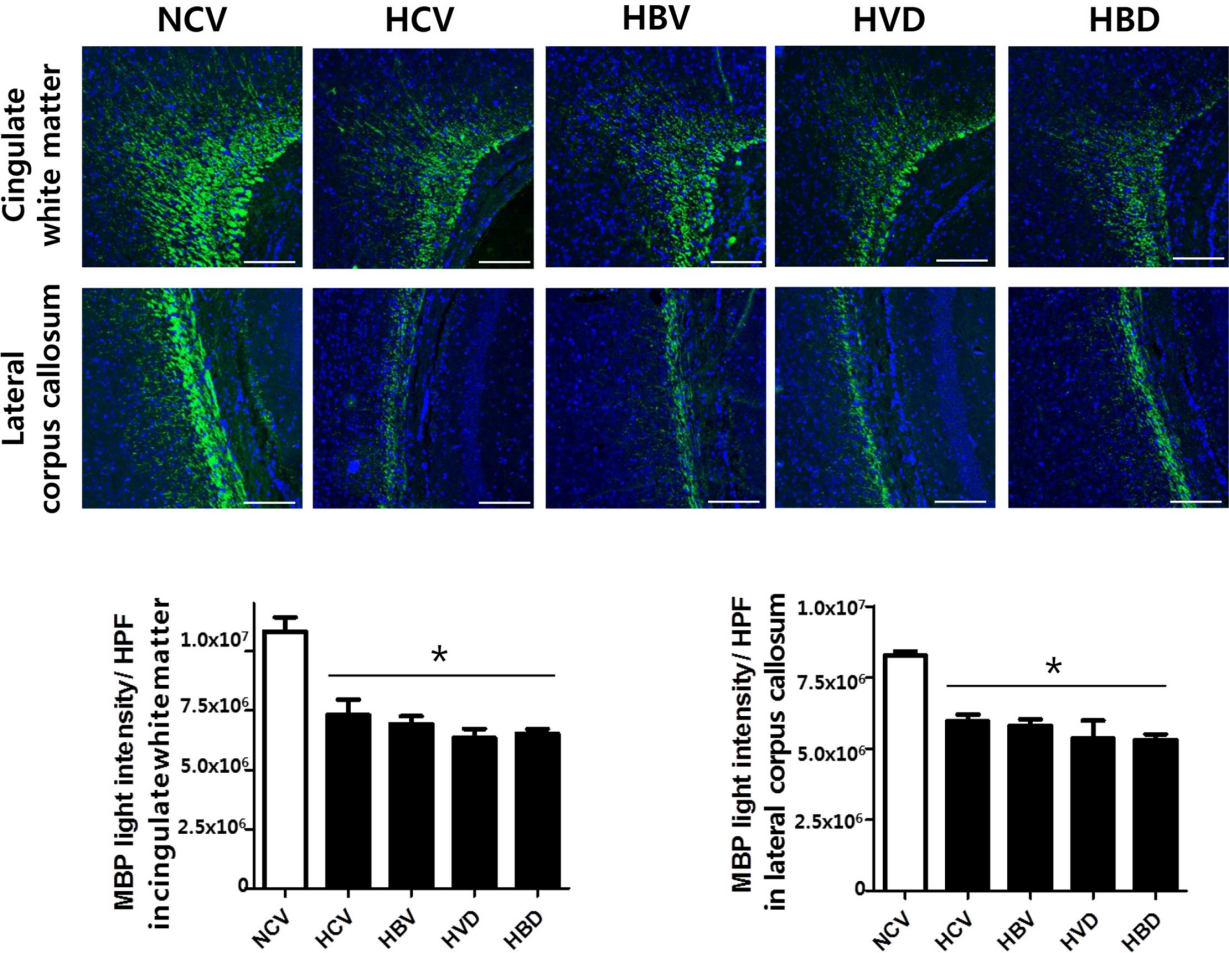
Myelination of brains exposed to hyperoxia and steroids. (A) Representative images of cingulate white matter stained with myelin basic protein (MBP; green) and with 4ʹ,6-diamidino-2-phenylindole (DAPI; blue) (scale bar, 200 μm), and (B) the average light intensity of MBP per high-power field (HPF) in cingulate white matter was measured at P14 at P14 (n = 6, 5, 6, 6 and 5 in NCV, HCV, HBV, HVD and HBD, respectively). Data are given as mean ± SEM. * *P* < 0.05 vs. NCV; # *P* < 0.05 vs. HCV; $ *P* < 0.05 vs. HBV. NCV, normoxia control with antenatal vehicle and postnatal vehicle; HCV, hyperoxia control with antenatal vehicle and postnatal vehicle; HBV, hyperoxia with antenatal betamethasone and postnatal vehicle; HVD, hyperoxia with antenatal vehicle and postnatal dexamethasone; and HBD, hyperoxia with antenatal betamethasone and postnatal dexamethasone.

Brain myelination was assessed by immunofluorescent staining for MBP in cingulate white matter and lateral corpus callosum at P14 (Fig 6A and 6B). Hyperoxic brain injury significantly reduced the density of MBP-positive nerve fibers in cingulate white matter and lateral corpus callosum at P14 (Fig 6A and 6B). MBP fluorescence intensity, indicative of the extent of brain myelination, was significantly reduced in the HCV, HBV, HVD, and HBD groups compared to that in the NCV group at P14.

### Oxidative stresses in lung and brain

**Fig 7:**
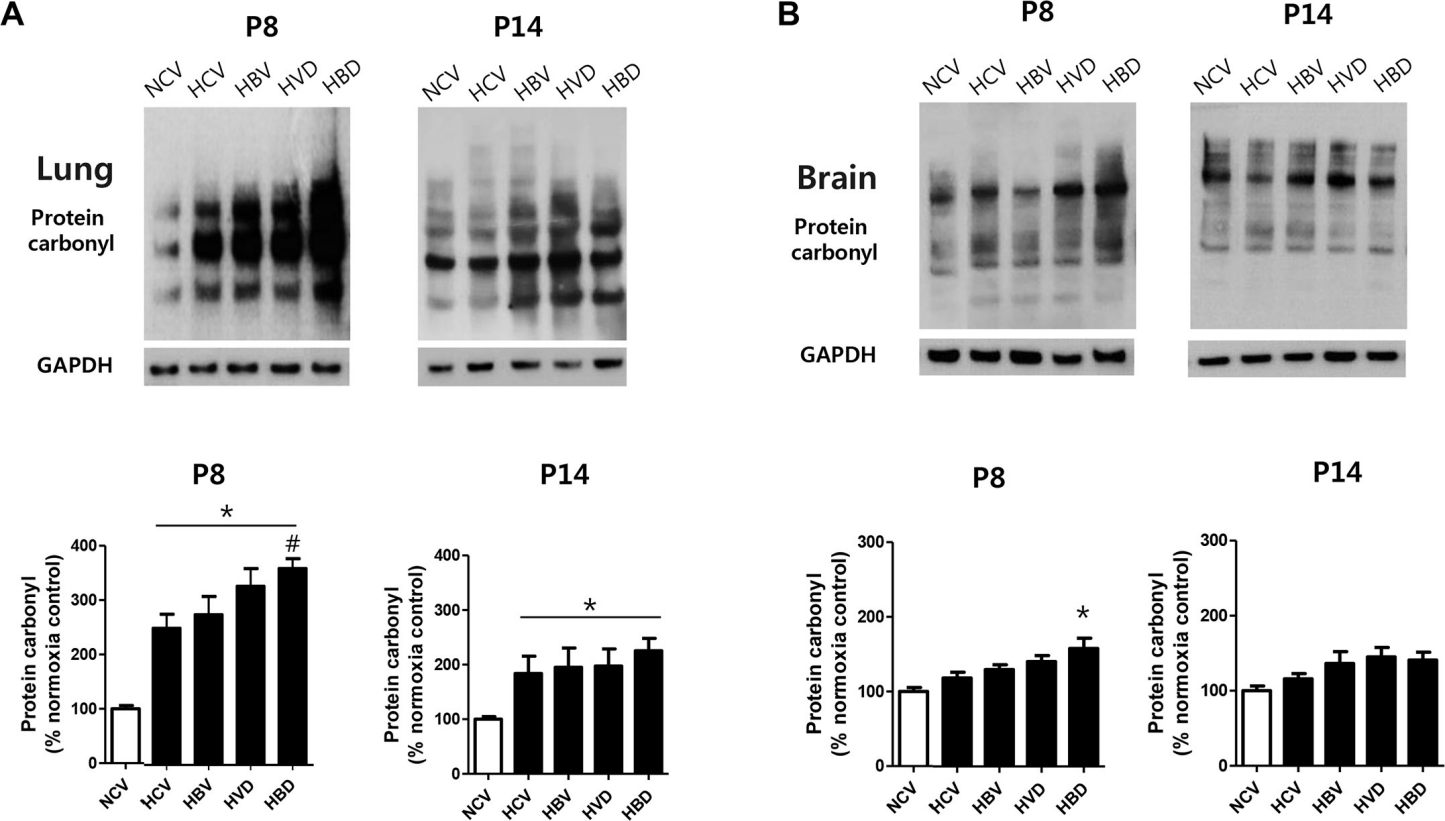
Western blotting to analyze lung and brain oxidative stresses. (A and B) Representative western blot for protein carbonyl and densitometric analysis of protein carbonyl contents normalized to GAPDH (loading control), given as percentage of normoxia control group, at P8 (n = 7, 6, 7, 6, and 7 in the NCV, HCV, HBV, HVD, and HBD groups, respectively) and at P14 (n = 6, 5, 5, 6 and 6 in NCV, HCV, HBV, HVD and HBD, respectively) rat lungs (A) and brains (B). Data are given as mean ± SEM. * *P* < 0.05 vs. NCV; # *P* < 0.05 vs. HCV. NCV, normoxia control with antenatal vehicle and postnatal vehicle; HCV, hyperoxia control with antenatal vehicle and postnatal vehicle; HBV, hyperoxia with antenatal betamethasone and postnatal vehicle; HVD, hyperoxia with antenatal vehicle and postnatal dexamethasone; and HBD, hyperoxia with antenatal betamethasone and postnatal dexamethasone.

Fig 7 shows the representative western blots and quantitative assays of protein carbonyl contents, a marker of oxidative stress, in the rat lung and brain at P8 and P14. While the protein carbonyl contents of the lung in HCV rats were significantly increased compared to those in NCV rats both at P8 and P14, the hyperoxia-induced increase in lung oxidative stress was significantly but transiently enhanced only in HBD rats but not in HBV and HVD rats at P8 but not at P14. Brain protein carbonyl contents were significantly but transiently increased only in the HBD group but not in the HCV, HBV, and HVD groups at P8 but not at P14.

### Correlation analyses of lung and brain injuries

**Fig 8:**
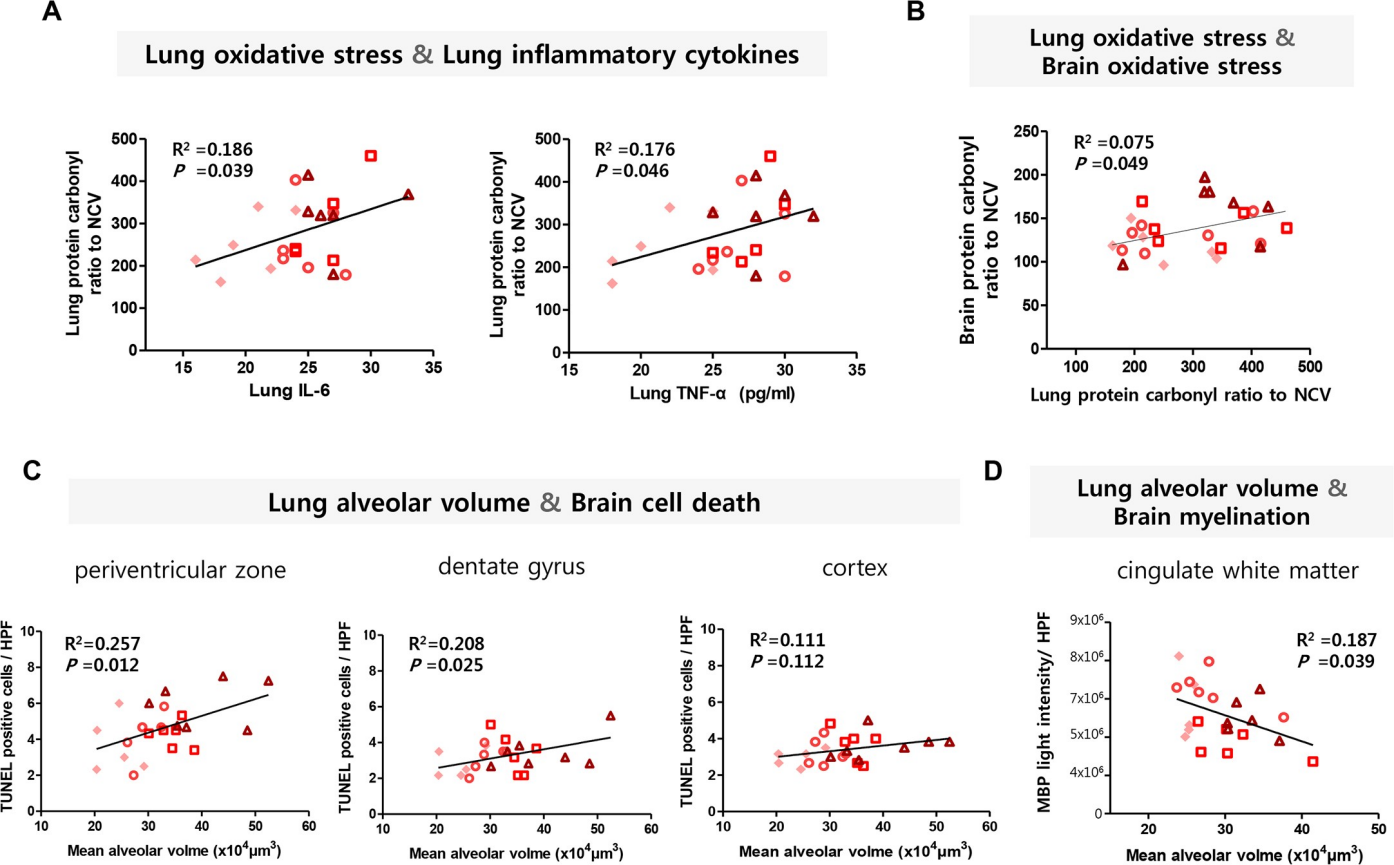
Correlation dot plots of lung and brain injury/or developmental levels. (A) Correlations between lung oxidative stress and lung inflammatory cytokines at P8. X-axis: lung inflammatory cytokine levels (pg/mL), such as IL-6 and TNF-α. Y-axis: contents (ratio to normal control vehicle, NCV) of lung’s protein carbonyl. (B–D) Correlations between lung and brain injuries. (B) X-axis: contents (ratio to NCV) of lung protein carbonyl at P8. Y-axe: contents (ratio to NCV) of brain protein carbonyl at P8. (C) X-axis: Mean alveolar volume (×10^4^μm^3^) at P8. Y-axis: number of TUNEL-positive cells per high-power field (HPF) in the periventricular zone, dentate gyrus and cortex at P8, respectively. (D) X-axis: Mean alveolar volume (×10^4^μm^3^) at P14. Y-axis: light intensity of myelin basic protein (MBP) in the cingulate white matter at P14. HCV, hyperoxia control with antenatal vehicle and postnatal vehicle; HBV, hyperoxia with antenatal betamethasone and postnatal vehicle; HVD, hyperoxia with antenatal vehicle and postnatal dexamethasone; and HBD, hyperoxia with antenatal betamethasone and postnatal dexamethasone.

In correlation analyses within hyperoxic groups, lung oxidative stress significantly correlated with lung inflammatory cytokines (R^2^ = 0.186, *P* = 0.039 for IL-6; R^2^ = 0.176, *P* = 0.046 for TNF-α) (Fig 8A) and brain oxidative stress (R^2^ = 0.075, *P* = 0.049) (Fig 8B). Lung alveolarization was significantly correlated with brain cell death (R^2^ = 0.257, *P* = 0.012 in the periventricular zone; R^2^ = 0.208, *P* = 0.025 in the dentate gyrus) and myelination (R^2^ = 0.187, *P* = 0.039) in the cingulate white matter (Fig 8C and 8D).

## Discussion

Developing an animal model that simulates the clinical BPD is very important not only for further delineating the pathophysiology and developing new and effective therapeutic modalities for BPD. In the clinical setting, survivors of BPD suffer from long-term injuries not only to the lung such as asthma or chronic obstructive pulmonary disease [2] but also to the brain including developmental delay and cerebral palsy [3–5]. Newborn rat models have several advantages such as low cost, fast turn-around time and low maintenance costs. Moreover, the saccular stage of the rat lung at birth is equivalent to that at 25–28 week of gestation in human lung development [19]. In our present and previous studies [6, 7], in addition to the well-known hyperoxic lung injuries such as impaired lung alveolarization and angiogenesis, we observed simultaneous brain injury as evidenced by retardation in brain weight gain, increased TUNEL- positive cells and reduced myelination, and a significant close correlation of lung and brain injuries in newborn rats. However, our model has limited capacity to fully simulate BPD in human preterm infants, because the newborn rats were born at term with short lung saccular period of 4 postnatal days, lack of mechanical ventilation to induce barotrauma and exposure to prolonged and severe hyperoxia is not currently common in preterm infants [12, 20, 21]. Moreover, multiple interventions to decrease the oxidative BPD injury in rodents have been shown to not work in humans [20]. Collectively, despite the main advantages, including hyperoxia exposure during the saccular to alveolar stages of lung development, given the limitations of the newborn rat model to appropriately simulate the clinical conditions of BPD in human preterm infants, the study results obtained from the rodent model cannot be directly extrapolated into humans, and extreme caution must be applied in the clinical translation of these data including the disease pathogenesis and therapeutic efficacy.

As BPD in preterm infants is a serious disorder that frequently causes mortality, the experimental design simulating this critical clinical condition is inevitably subject to the comparison of mortality among the study groups for precluding distortion of the experimental data. However, as the use of mortality as an endpoint are somewhat opposed to an ethics viewpoint in animal experiment, further studies will be necessary to develop an animal model that could utilize alternative method to assessment of mortality regarding this issue for the animal welfare.

In this study, postnatal dexamethasone alone, but not antenatal betamethasone alone significantly enhanced the hyperoxia-induced reduction in body and brain weight gain, impaired lung alveolarization and angiogenesis, and increased the number of TUNEL-positive cells in both the lung and brain. However, the co-administration of antenatal betamethasone significantly exacerbated the detrimental effects of postnatal dexamethasone on hyperoxia-induced impaired lung alveolarization and increased brain TUNEL-positive cells. Our data showing the additive detrimental effects on hyperoxic lung and brain injuries with co-administration of antenatal betamethasone and postnatal dexamethasone suggest that extreme caution must be taken when clinically using these drugs together in preterm infants to improve mortality and prevent BPD.

The mechanisms through which postnatal dexamethasone alone and co-administration of antenatal betamethasone additively exert detrimental effects on hyperoxic lung and brain injuries remain unclear. In this study, the hyperoxia-induced increase in lung and brain oxidative stress and lung inflammatory cytokines were significantly enhanced by the co-administration of antenatal betamethasone and postnatal dexamethasone. Moreover, significant correlations between lung oxidative stress and lung inflammatory cytokines and brain oxidative stress, as well as between lung MLI and brain cell death and myelination were observed in this study. Collectively, our data suggest that the promotion of hyperoxia-induced oxidative stress [22] and ensuing aggravation of the inflammatory responses play a major role in the additive detrimental effects of co-administered antenatal betamethasone and postnatal dexamethasone on the hyperoxic lung and brain injuries in newborn rats.

In the present study, the 0.2 mg/kg dose of antenatal betamethasone administrated at E19 and E20 was chosen to minimize its effects on fetal mRNA and protein levels [12, 23]. However, as rat E19–20 corresponds to gestational age of 20–22 weeks in humans [20], the timing of the administration might be too early to simulate the beneficial effects of clinical antenatal steroid use, which are usually not observed before 23 weeks of gestation. Furthermore, as an exposure period of 48 h in the rat corresponds to 10% of gestation, which is too long compared with that in humans (0.8% of gestation), the relatively prolonged exposure to antenatal betamethasone might be another limitation of the rodent model. We chose the same tapering dose of dexamethasone (0.5, 0.3 and 0.1mg/kg at P5, 6, and 7, respectively) that is used commonly in clinical practice to prevent BPD in the premature infants in this newborn animal study [12, 24]. However, as 3 postnatal days in rats corresponds to 3 weeks in humans, the relatively prolonged exposure to dexamethasone might be another limitation of the rodent model. Further studies are necessary to develop more clinically relevant dose and timing regimens for both antenatal and postnatal glucocorticoids in the animal models of BPD.

Glucocorticoids such as dexamethasone are one of the most potent anti-inflammatory agents [25] and, thus, are often used clinically to prevent BPD in premature infants [26]. Our data showing the promotion of oxidative stress and ensuing pro-inflammatory effects of postnatal dexamethasone and co-administered antenatal betamethasone are in direct contrast to the classical anti-inflammatory responses expected after perinatal steroid treatment [27] and remain difficult to explain. In concordance with our data, dexamethasone pretreatment increased hyperoxic lung inflammatory responses in male Sprague-Dawley rats [28], and chronic steroid exposure facilitated the paradoxical pro-inflammatory responses to injury in the brain [29, 30]. Collectively, these findings suggest that classifying perinatal steroids as strict anti-inflammatory agents is an oversimplification. Therefore, further studies are necessary to elucidate the precise mechanisms and determinants of the contradictory pro- and anti-inflammatory effects of perinatal steroids including the timing and dosage for their future successful clinical translation with optimal efficacy and safety.

In summary, prolonged exposure to hyperoxia resulted in hyperoxic lung injuries, as evidenced by impaired lung alveolarization and angiogenesis, and brain injuries, as evidenced by reduced brain weight gain and myelination and an increased number of TUNEL-positive cells. While postnatal dexamethasone alone, but not antenatal betamethasone alone, significantly aggravated hyperoxic lung and brain injuries, the co-administration of antenatal betamethasone significantly exacerbated the detrimental effects of postnatal dexamethasone on hyperoxic lung and brain injuries primarily through the promotion of oxidative stress and the ensuing inflammatory responses in newborn rats.
